# Introducing Interesting Images

**DOI:** 10.3390/diagnostics5030294

**Published:** 2015-07-09

**Authors:** Andreas Kjaer

**Affiliations:** *Editor-in-Chief of Diagnostics*, Department of Clinical Physiology, Nuclear Medicine & PET, National University Hospital, Rigshospitalet, University of Copenhagen, Blegdamsvej 9, DK-2100 Copenhagen, Denmark; E-Mail: akjaer@sund.ku.dk; Tel.: +45-3545-4216

Since the launch of *Diagnostics,* a major part of contributions have been within the field of medical imaging. For researchers and clinicians working within this rapidly evolving field, e.g., with positron emission tomography (PET), computed tomography (CT), magnetic resonance imaging (MRI), ultrasound or other imaging modalities, difficult cases or unsuspected findings often represent a diagnostic challenge. In such cases a search for similar images described in peer-review journals is an integral part of the work-up. Therefore, at *Diagnostics* we recently introduced a new type of submission named *Interesting Images*. The format is intentionally well suited for rapid communication and a short turn-around time in the peer-review process. We encourage submission within this category. The number of images included is in no way limited and is at the discretion of the authors. Where dynamic data are of relevance, videos may be added as supplementary files. No regular text, *i.e*., introduction/methods/results/discussion, is needed. Instead, the images should be accompanied by a detailed legend with no restriction in length describing the patient case and image findings, as well as interpretation. Further, an unstructured abstract should be included as well as key words and references.

It is our hope, that these cases will be viewed and used by everybody working with medical imaging and image interpretation. Since *Diagnostics* is fully *open access*, the Interesting Images will be freely available to all. As an example of a clinical case, PET images of so-called brown tumors, FDG-avid benign, osteolytic lesions often misinterpreted as bone metastases, were recently published in *Diagnostics* [1].

Also images not related to clinical work-up of patients are encouraged as submissions if they represent important and novel information. As an example in this category, we recently published a case of a dog with a spontaneous tumor where the new concept of *hyperPET*, the combination of PET and magnetic resonance spectroscopic imaging of hyperpolarized ^13^C containing compounds, demonstrated that fluorodeoxyglucose (FDG)-PET may not always be a reflection of the *Warburg effect* [2].

It is our hope that this new submission category will be well received among researchers and clinicians and that they will send in high quality contributions. As always, we can on our side promise a fast handling and review procedure followed by prompt publication that is fully *open access*.
